# Evaluation of the Risk of Getting Peripheral Artery Disease in Rheumatoid Arthritis and the Selection of Appropriate Diagnostic Methods

**DOI:** 10.7759/cureus.9782

**Published:** 2020-08-16

**Authors:** Surik Sedrakyan, Tehreem Fatima, Mst. Khaleda Khatun, Muhammad R Awan, Nkechi A Okam, Nusrat Jahan

**Affiliations:** 1 Internal Medicine, California Institute of Behavioral Neurosciences and Psychology, Fairfield, USA; 2 Internal Medicine, California Institute of Behavioural Neurosciences and Psychology, Fairfield, USA; 3 Internal Medicine, California Institute of Behavorial Neurosciences and Psychology, Fairfield, USA

**Keywords:** rheumatoid arthritis, peripheral artery disease, cardiovascular disease, risk factors, arterial stiffness, endothelial dysfunction, vascular obstruction diagnosis

## Abstract

This study aims to review the evidence regarding the association between peripheral artery disease (PAD) and rheumatoid arthritis (RA), as well as influential underlying factors and diagnostic options. Eligible literature was searched in PubMed published up to June 1, 2020, in English. Case studies, case series, reviews, and meta-analyses were excluded. We also excluded non-human studies and those 20 years and older. A total of 44 studies were finally incorporated in the narrative review. The results indicated that compared to controls, RA patients are more prone to PAD. Traditional risk factors, disease-characteristics, vitamin D deficiency, therapy used, and other relevant conditions have a variable effect on overall PAD progression. Studies comparing diagnostic options revealed that vascular function and morphology are connected but are still distinctive processes. In early-stage disease, there are functional alterations in the endothelium that can be controlled by anti-inflammatory medications. Ankle-Brachial Index (ABI) <0.9 might not be quite susceptible to PAD evaluation. Supplemental diagnostic tools could detect vascular disease in the preclinical stage. Most risk factors are adjustable, and the management will have a good impact on vascular health. PAD is mostly subclinical when the therapeutic options have a better impact. Diagnostic modalities should be chosen depending on the features of RA. In addition, multiple diagnostic options increase the accuracy of PAD diagnosis. Future prospective studies with larger populations at different age groups and different disease activity duration are essential to make firm conclusions and better understand the phenomenon of RA and PAD.

## Introduction and background

Rheumatoid arthritis (RA) is one of the most commonly diagnosed systemic inflammatory disorders, which primarily involves synovial joints. RA is also associated with systemic and nonarticular manifestations, which share similar pathomechanisms with the inflammatory process in joints [1]. It is stated that the incidence of cardiovascular (CV) disease is much higher in RA patients than in the general population [2]. Some factors impair vascular health and integrity. People with RA have more prevalence of traditional risk factors such as hypertension, dyslipidemia, smoking, and diabetes mellitus [3-6]. An excess of traditional risk factors increases the probability of CV disease formation. Besides, a systemic inflammatory state and dysregulative immune processes cause endothelial damage [7,8]. Endothelial damage is a continuous process, with a balance between the magnitude of injury and the capacity for regeneration. Long-standing oxidative stress, aging, exhausted endothelial progenitor cells (EPC), and other detrimental processes impair the endothelium’s regenerative capacity, resulting in endothelial dysfunction (ED). Endothelial dysfunction is a primary mechanism of premature atherosclerotic changes in vascular beds [3]. During prolonged inflammatory states, cytokines, inflammatory chemicals, activate endothelial cells (ECs) phenotype. ECs then produce the cytokines interleukin 1 (IL1) and interleukin 8 (IL8), and express the surface molecules intercellular adhesion molecule 1 (ICAM-1), vascular cell adhesion protein 1 (Vcam-1), and cluster of differentiation 40 (CD40) [9]. Hence, endothelial cells enter a pathological cycle. As ED is a change of normal physiology of EC, the endothelium becomes more prone to vasoconstriction. The switch also induces prothrombotic and proliferative features. ED is a functional deterioration of the endothelium. With the progression of the disease, morphological alterations arise [10,11]. ED emerges differentially in vascular beds, making particular regions more inclined to atherosclerosis formation [12]. That is why each vascular bed requires a unique approach. In the presented literature, we will discuss mainly the subgroup of CV disease known as peripheral artery disease (PAD). Compared to coronary artery disease (CAD) and cerebrovascular disease, PAD and RA association is less appraised and explored. Arthritis in the lower limb joints impedes walking and causes significant discomfort. People with PAD experience a hard time walking, climbing stairs, or performing other regular activities. Coexisting problems decline the functional status of the patients. One study showed that abnormal Doppler and Toe-Brachial Index (TBI) findings are associated with slower walking velocity [13]. Moreover, slow gait speed is a risk factor for persistent and severe lower limb restriction, hospitalization, and mortality [14]. Assessing RA and PAD association as a separate vascular bed will allow us to determine which factors affect PAD formation and which subgroup of RA patients are inclined to get peripheral artery disease. Risk stratification is the first step to manage PAD. Secondly, a proper diagnostic method is desirable. There are several diagnostic options that investigate ED, arterial stiffness, vessel obstruction, or other relevant abnormalities. If we can evaluate which diagnostic options have more sensitivity to anticipate PAD, vascular changes might be detected in the early stages. Recording of vascular changes in the preclinical stage allows fewer interventions and greater benefits. Furthermore, false-negative results take away a crucial preclinical period when preventive and therapeutic actions will have the most impact. The current narrative review aims to assess the most significant risk factors and their impact on PAD in RA as a separate vascular unit and which available diagnostic modalities might find peripheral vascular changes in its early, preclinical form.

## Review

Methods

We included studies published before June 1, 2020, from PubMed. Regular keywords and Medical Subject Headings (MeSH) subheadings were used for data collection (Tables 1, 2).

**Table 1:** Represents MeSH keywords used to find appropriate articles for the narrative review. MeSH: Medical Subject Headings

MeSH keywords	Rheumatoid arthritis (subheading-Peripheral artery disease)	Rheumatoid Arthritis subheading- (Arterial disease diagnosis)
Total records	87	853
Records selected	58	506

**Table 2:** Represents regular keywords used to find appropriate articles for the narrative review. RA: rheumatoid arthritis

General keyword	RA and endothelial dysfunction	RA and arterial stiffness
Total records	745	203
Records selected	294	140

Studies were selected after applying the following inclusion/exclusion criteria:

Inclusion Criteria

1. Paper published within the past 20 years

2. Studies including only humans 

3. Paper published in the English language

4. The study types were cohort, case-control, cross-sectional studies, and literature reviews.

Exclusion Criteria

1. Animal Studies

2. Non-English language literature

3. Case reports and case series studies

4. Systematic reviews and Meta-analysis studies

To begin with, a total of 1888 studies were involved; after applying primary filters we were left with 998 articles (Table 3).

**Table 3:** Represents the total number of literature selected after applying the inclusion/exclusion criteria. RA: rheumatoid arthritis, PAD: peripheral arterial disease

	RA and PAD	RA and arterial stiffness	RA and arterial disease diagnosis	RA and endothelial dysfunction	Total
Total records	87	203	853	745	1888
Inclusion/exclusion					
Published in the last 20 years	77	199	708	637	1621
Humans only	77	170	706	534	1487
English literature	75	165	643	487	1370
Excluding reviews, meta-analysis and case studies	58	140	506	294	998

We screened the rest of the articles by the titles and abstracts. If a decision could not be made from titles and abstracts, full articles were retrieved. Articles were removed because they lacked the outcome of interest or were duplicates.

The outcomes of interest were peripheral artery disease associated with RA, including lower limb occlusive disease, leg ulcers, abdominal aortic aneurysm, evaluation of risk factors, diagnostic options for peripheral vasculature, as well as endothelial dysfunction and arterial stiffness.

Results

Finally, after a refined manual review, we were left with 44 articles. The data included:

· Case-control - nine studies

· Cohort - 12 studies

· Cross-sectional - 19 studies

· Narrative review - one study

· Clinical trial - three studies

The maximum number of subjects in the studies was 69,755 [15] and the minimum was 41 [16]. The total number of subjects included in all 44 studies was 108,280, from which 49,142 were people with diagnosed RA.

**Figure 1:**
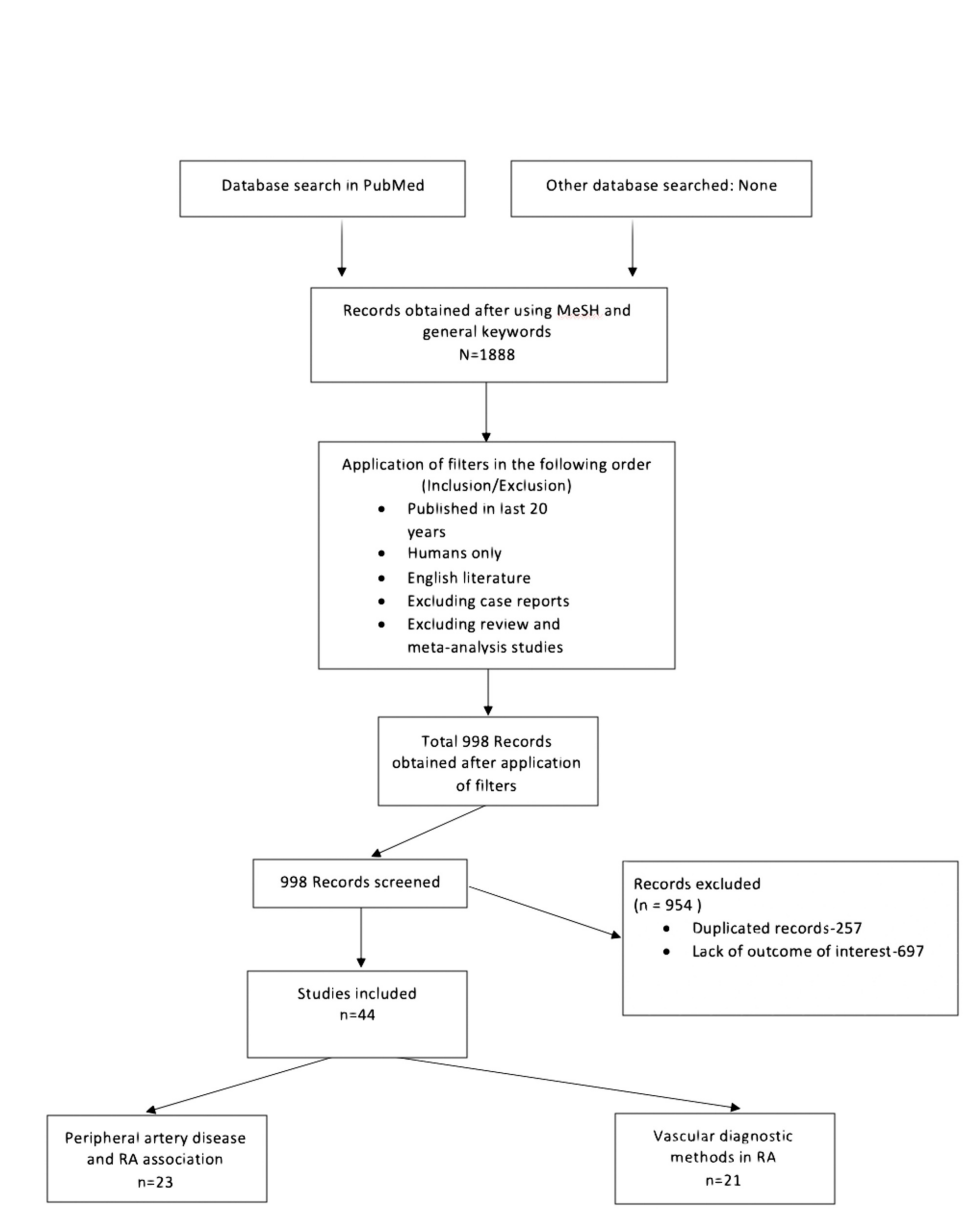
Represents a flowchart of the selection process of the current narrative review. RA: rheumatoid arthritis, MeSH: Medical Subject Headings

Figure 1 below shows the flowchart with the process of the current literature review.

Discussion

The narrative review, by the analysis of 44 articles, found the relation of PAD to RA. We documented that many conditions were independently related to a higher risk of PAD. Several diagnostic options were found to be useful as a marker of vascular disease. Each diagnostic parameter appears to have specific indications and usability. In each pathological condition, different factors have an impact on ED [3]. Cardiovascular disease risk factors should be estimated and controlled separately in RA patients. There is a higher prevalence of several traditional risk factors among the RA population. Higher systolic and diastolic blood pressures (SBP/DBP) are seen in RA [3-6,10,17]. In several studies, higher SBP was associated with higher arterial stiffness [6,18-20]. Higher arterial stiffness correlated with elevated nighttime SBP and the non-dipping pattern [21]. In addition, higher levels of triglycerides, total cholesterol, low-density lipoprotein (LDL), glucose, and increased body mass index (BMI) were seen in our discussed population [3,5,6,13,22,23,24]. Many studies ruled out smokers from study groups because of the evident vasoconstrictor effect, and only some studies showed a higher prevalence of smokers [5,6]. Not only current smoking but also former smoking harms the vasculature [25]. Inflammatory markers might partially induce higher prevalence of these conditions; tumor necrosis factor-alpha (TNF-alpha), interleukin 10 (IL10), interleukin 6 (IL6), and C-reactive protein (CRP) are correlated with SBP, and CRP, von Willebrand factor (VWF) have a correlation with triglyceride level [6]. In almost all studies, erythrocyte sedimentation rate (ESR) and CRP are significantly higher in RA patients, including high inflammatory and normal inflammatory status patients. Vitamin D level was lower, and VWF was higher in the people with RA disease [6,26,27].

Traditional Risk Factors

Prevalence does not necessarily mean causation. High SBP, DBP, total serum cholesterol, LDL, BMI, insulin resistance, and smoking are related to vascular morphological characteristics, measured by pulse wave velocity (PWV), augmentation index (Aix), and ankle-brachial Index (ABI) [5,6,18-20,23,25]. ED is associated with SBP, LDL, smoking, and age [2,24,28]. We found no other clinically significant association of traditional risk factors in our database. With aging, increased collagen deposition in the vessel wall makes it stiffer. Mostly encompassing central arteries, central systolic blood pressure increases. A prolonged inflammatory state includes peripheral arteries in the process. A controversial and notable finding is Ya-Wen Chuang’s study, where over 30,000 people were studied for peripheral artery occlusive disease [19]. The outcome was that people with early-stage disease and age <49 had a higher chance of getting peripheral arterial occlusive disease (PAOD). Insulin resistance induced by immune dysregulation is the primary mechanism of hyperglycemia, a condition associated with arterial stiffness [6,25]. Lipid metabolism abnormalities are associated with both ED and arterial stiffness. Dyslipidemia takes part in both atheroma formation and the early stages of atherosclerosis [26]. 

Inflammatory Status

Almost all studies concluded that systemic inflammation affects endothelial dysfunction and arterial stiffness. A study involving 5638 patients found that 30% of CV disease was contributable to RA characteristics. The limitation of the study is that it evaluated CV disease as a whole, not a particular bed [8]. Flow-mediated dilation (FMD) was found to be inversely correlated with disease activity score in 28 joints (DAS28), The Health Assessment Questionnaire (HAQ), inflammatory markers, IL6, TNF-alpha, and positively correlated with EPC% [29]. Sule Gunter’s study showed that arterial stiffness measured by several parameters was associated with ESR and CRP [20]. One controversial study is Fenling Fan and co-workers’ finding, that systemic inflammation does not contribute to ED [6]. However, there are some limitations. At first, the usage of anti-inflammatory drugs was not adjusted, and the inflammatory markers did not reach clinical significance. There is a predisposition of thrombosis in high DAS28 and CRP level RA patients, counted with rotational thromboelastometry (ROTEM) [30]. ESR and CRP levels are associated with functional and morphological changes. Longitudinal use of anti-inflammatory medications improves only microvascular and macrovascular endothelium-dependent functions, but not vascular morphology [31]. So modulation of ED does not necessarily modify arterial stiffness [11]. Arterial stiffness is an irreversible process, aggravated by an inflammatory state. Anti-inflammatory medications do not eradicate irreversible changes, but decrease further arterial stiffness progression. Rheumatoid Factor positivity is associated with more morphological changes in the vasculature [20,32]. A study headed by Laura Geraldino-Pardilla used F-Fluorodeoxyglucose positron emission tomography/computed tomography (F-FDG-PET/CT) to detect ascending aorta inflammation [7]. F-FDG-PET/CT diagnostic option is gainful but expensive. Anti-citrullinated protein/peptide antibody (ACPA) seropositivity was independently associated with inflammation. Consequently, immune dysregulation, possibly, plays a role in PAD progression. In contrast, Gian Luca Erre and co-workers found an association between peripheral ED and ACPA negativity [28]. Immune dysregulation role and ACPA association warrant further exploration. 

Medication Effects

TNF-alpha and IL6 are key inflammatory cytokines in RA [33]. Anti-TNF-alpha therapy has a valuable effect on vascular homeostasis. It decreases the inflammatory process directly by decreasing inflammatory cytokines and reactive oxygen species levels. An additional effect is a proportional reduction of circulating CD4+CD28null cells. A higher percentage of CD4+28null cells are independent contributors for atheroma formation [22]. Treatment with methotrexate alone or with the combination of other disease-modifying anti-rheumatic drugs (DMARDs) demonstrates a reduction of central SBP, DBP, and PWV [34]. In one study, six months of methotrexate therapy increased nitric oxide (NO) bioavailability [acetylcholine (Ach) response] but not the sensitivity of vascular smooth muscles to NO [sodium nitroprusside (SNP) response] [17]. Statin supplement is expected to be beneficial in RA. It reduces the production of inflammatory molecules and disrupts the oxidative stress/inflammation cycle [35]. Tetrahydrobiopterin (BH4) is a cofactor of nitric oxide synthase. Short-term supplementation of BH4 improves ED, but no effect was seen on arterial stiffness [36]. These drugs generate vasculoprotective effects and have the potential to be repurposed for RA patients. Glucocorticoids are popular among RA patients and can reach high cumulative levels. In otherwise healthy patients, chronic use of glucocorticoids has a detrimental effect on the endothelium. The process is different in inflammatory states. Glucocorticoids are known for their anti-inflammatory properties, and in low doses, supposedly, could even have an antiatherogenic effect, provided the inflammatory nature of atherosclerosis [37]. The beneficial effect on the endothelium might be due to decreased expression of surface inflammatory cytokines (IL6, TNF-alpha). However, common belief is the harmful effect of glucocorticoids on endothelial function. From the scarce and controversial studies available most showed no effect on the endothelium with long term glucocorticoid use. Nevertheless, because of heterogeneity outcomes, studies are inconclusive. Interestingly, the duration of exposure seems not to have an impact on endothelial function [18,37]. On the other hand, the cumulative glucocorticoid dose is associated with arterial incompressibility defined as ABI>1.3. No significant association was found between lower extremity obstruction and glucocorticoid use. Glucocorticoid-induced calcification seems to be the sole mechanism of higher arterial incompressibility [38]. Cortical bone loss is associated with arterial incompressibility. Thus, if specialists find bone changes among RA patients, they need to worry about arterial lesions and vice versa. These two findings are separately associated with glucocorticoid use [25].

Vitamin D Deficiency

Albeit in general population 25(OH) vitamin D deficiency is prevalent, in RA patients, the deficiency is more expressed. Moreover, in high inflammatory states, vitamin D level is much less than in mild inflammatory states [26]. Vitamin D plays an influential role in immune regulation, insulin sensitivity, and cardiovascular system. Low vitamin D level is associated with the loss of immune tolerance and the presence of autoimmune processes. In addition, 25(OH) vitamin D plays a role in balancing RA disease activity by modulating inflammatory cytokines expression. Lower 25(OH) vitamin D level is associated with more morning stiffness, higher DAS28 score, and higher HAQ score. Vitamin D deficiency also indirectly affects endothelial function by eliciting insulin resistance [27]. Vitamin D supplementation improves endothelial function, measured by FMD. Interestingly, the effect on ECs is more significant in higher inflammatory state patients [26]. Nonetheless, longitudinal studies are needed to certainly assess the benefits of vitamin D supplementation to manage endothelial function.

NO synthesis inhibitors

Symmetric (SDMA) and asymmetric (ADMA) dimethylarginines are considered a novel biomarkers of CV disease, including in RA patients [39]. ADMA and SDMA dysregulated metabolism have an inhibitory impact on NO synthesis. In high inflammatory states, ADMA:SDMA ratio and SDMA level are negatively associated with microvascular endothelial-dependent Ach, independent SNP functions, and arterial stiffness. No association was found between normal inflammatory states and macrovascular function [39]. ADMA and SDMA metabolism impairment not only initiates endothelial damage in early stages but also plays a role in the later development of atherosclerosis in high inflammatory conditions [39,40]. These studies indicate possible individual roles of ADMA and SDMA in vascular homeostasis derangement among RA patients. Targeting ADMA and SDMA specific pathways should be considered in the future for adjusting CV disease.

Homocysteine

Homocysteine is considered a biomarker of vascular function, but causality is not yet proven. Homocysteine amount is generally higher in RA patients, but some studies showed not constant increase in the homocysteine level among RA patients mostly because of B6 supplements [10]. Adequate intake of B vitamins might prevent homocysteine elevation in RA patients. Clinical trials are needed to evaluate B6 supplementation for vascular function. Resting blood flow is inversely correlated with homocysteine in RA patients. It may be connected to decreased NO bioavailability and a higher rate of oxidative stress. In addition, Inflammatory factors contribute to higher homocysteine levels. Vascular function could be affected by homocysteine, and a hyperinflammatory state intensifies the effect [10]. 

Genotype

RA patients with particular genetic factors such as Human Leukocyte Antigen DRB1 beta chain (HLA DRB1) specific genotypes are more prone to ED and resulting accelerated atherosclerosis [25,41]. If confirmed by prospective studies, genotype status might be a predictor of future cardiovascular events in RA. 

Diagnostic Options and Parameters

It is precious to determine the association of endothelial dysfunction and arterial stiffness. Acknowledging whether they are related to each other or are independent processes will somewhat clear uncertainty in atherosclerosis genesis. Many studies suggested no association between microvascular and macrovascular changes in RA and healthy populations. The processes are going independently [36]. The only significant finding was that macrovascular endothelium-independent function is associated with carotid atherosclerosis. These two findings reflect smooth muscle dysfunction: hypertrophy and decreased relaxation [11]. In comparison, another study found a moderate association between them [31]. They explained it by the fact that diagnostic tools have an effect on different types of receptors, and NO is not the only vasodilatory mediator. In involved studies, microvascular ED was mostly measured using Laser Doppler imaging with iontophoresis of 1% Ach (endothelium-dependent) and 1% SNP (endothelium-independent). Assessment of macrovascular endothelial-dependent function was performed using FMD and endothelium-independent function by administering glyceryl trinitrate tablets [2,17]. In endothelial dysfunction, there are only functional changes compared to arterial stiffness, where mechanical and functional changes play a huge role. Mechanical lesions follow functional abnormalities; accordingly, there is a time lag between these two processes [29]. Microvascular function is affected earlier than the macrovascular one in RA individuals. Importantly, arterial stiffness occurs only in medium-large vessels. Thus these two parameters could not be used interchangeably, and examination of endothelial function should incorporate morphological and functional appraisal [10].

ABI Measurement

One of the most utilized diagnostic options is ABI measurement. In the general population, it is considered a superior option for its simplicity, non-invasiveness, and is very easy to determine routinely [42]. Prevalence of arterial obstruction, diagnosed as ABI<0.9, was 15-16% in several studies [26,38,43]. The study population had no previous history of CV disease and had at least five years diagnosed RA. Interestingly, in J. K. Alkaabi’s study, only 30% had symptoms (mostly claudication, less common rest pain, and ulcer) [4]. The latter displays that clinical and instrumental diagnoses of PAD do not match. People with RA often lead a sedentary lifestyle because of musculoskeletal impairment and do not complain of exercise-induced ischemic symptoms [2]. A cohort study suggested that the 30-year cumulative effect of RA on PAD was 19.6% presented as renal artery stenosis, abdominal aorta aneurysm (AAA), peripheral artery atherosclerotic disease, and thromboembolic events. Moreover, only peripheral artery atherosclerotic disease incidence was 16.1%. Six hundred nine RA patients were included in this study. For detecting peripheral artery obstructive disease, they used mostly ABI<0.9 parameter. Another study explored artery disease by ABI and Doppler spectral waveform analysis [44]. From 100 participants, 30% had biphasic waveform finding (subclinical process), but only 4% had ABI<0.9. This finding underscores the fact that the prevalence of the subclinical disease is much higher. ABI measurement alone will not be complete for PAD detection. Exercise-induced ABI measurement reveals some of the covert abnormalities. People with risk factors and normal ABI need further examination with ABI after exercise, which is useful in identifying patients with increased mortality rates [42]. One study, including only Koreans, found the prevalence of PAD to be just 1.5% [18]. They measured ABI and brachial-ankle PWV. This large difference in PAD prevalence among Koreans may be explained by the fact that Asians have lower PAD rates [45]. The review showed that healthy or diabetic Asians have a significantly lower prevalence of PAD than white and black ones. Besides, ED was not measured here. A leg ulcer is a common manifestation of PAD. Several underlying conditions may predispose RA patients to leg ulcer formation [16]. Another study showed that the majority of leg ulcers found in RA patients are of arterial/venous origin [33]. Ora Shovman and co-workers’ study that included 69,755 subjects discusses AAA association with RA [15]. They found an independent association between AAA and RA, after adjustment of several risk factors [OR (odds ratio) 1.406, 95% CI 1.094-1.789; p = 0.006]. The latter confirms the previously mentioned study’s findings [2]. Arterial incompressibility, often found in RA patients, is a stiffening of the vascular wall. The primary mechanism is a vessel’s medial layer calcification. Although incompressibility increases CV complications risk, it is a distinct process with distinguishable risk factors. ABI>1.3 is sensitive and specific enough to detect arterial calcification (88% and 80%, respectively) [46]. From 234 patients enrolled, 20% had at least one calcified artery. 

Arterial Stiffness

Aging and CV risk factors cause aortic elastin fibers to be replaced by collagen, resulting in arteriosclerosis. Arteriosclerosis can occur in the absence of atherosclerosis. Arteriosclerosis causes arterial stiffness, which is mostly measured by pulse wave velocity (PWV). PWV is estimated by measuring the pulse wave at two sites in the vasculature and estimating the wave’s path length. While PWV is a direct measure of large arterial stiffness, the augmentation index (Aix) is a surrogate marker of arterial rigidity. It can be affected not only by the properties of large arteries but also by the ventricular ejection and peripheral hemodynamics. Although Aix was primarily used for wave reflection measurement, it is also suggestive of arterial compliance and reservoir function. There is a limitation to Aix as a measure of wave reflection [47]. This study did not involve people with hypertension, but still, the finding is worthwhile. Reflected wave pressure still can be measured and be useful for detecting plaques. Another study showed that reflected wave pressure of 25mmhg could predict plaque presence by sensitivity, specificity, positive predictive value, and negative predictive value of 45.2%, 89.3%, 78.6%, and 66.2%, respectively [48]. Nevertheless, the Aix parameter can now easily be calculated together with PWV with the use of sphygmoCor technology. Separate Aix measurement might be non-conclusive.

Femoral IMT

Not many studies included femoral intima-media thickness (IMT) in vascular health disease evaluation. One study suggested that the presence of RA is an independent risk factor for increased femoral IMT [49]. IMT shows to be a marker of peripheral artery disease.

Early-Stage RA

In early-stage RA disease, assessing ED for detecting subclinical peripheral artery disease might be enough. No peripheral morphological changes were found in short RA [50]. On the contrary, Aamer Sandoo discovered that aortic PWV was higher in people with early-stage RA than non-RA controls [31]. Physiologically BP differs in the arterial tree. Central arteries are highly elastic, while brachial arteries are much stiffer. It is documented that in early-stage disease, the aorta is more vulnerable to central systolic hypertension and inflammatory mediators [20,43]. Aortic PWV and Aix are most frequently investigated central hemodynamic measures. There is not any non-invasive option for measuring central BP and pulse pressure. Young to middle-aged RA patients free from CV risk factors and in mild inflammatory states are still under the risk of having ED (FMD measurement), as stated in one study [24]. Another study showed no difference in RHI in younger patients with mild inflammation compare to healthy individuals [3].

Limitations

This narrative review involved only PubMed derived articles; thus, some relevant articles may be missed. We included only peripheral artery measurements, excluding carotid IMT, a sensitive tool for carotid atherosclerosis detection. We also excluded case and case series studies, meta-analysis, and systematic reviews. Only human and <20-year-old studies were involved.

## Conclusions

Our analysis of all published literature concluded that PAD is a multifactorial process in people with RA. Traditional risk factors are documented to be related to both ED and arterial stiffness. Management of controllable risk factors will have a good impact on vascular health. The systemic inflammatory state is an independent contributor to ED and arterial stiffness formation. Inflammation regulation improves micro- and macrovascular ED, but no amelioration is noticed in morphological processes. Arterial stiffness seems to be an irreversible process. ADMA:SDMA has a possible individual role, mostly seen in high inflammatory states. Anti-TNF, methotrexate, and statins showed strong vasculoprotective effects. Use of these medications facilitates the disease process and, at the same time, decreases the chances of further occurrence. The effect of glucocorticoids remains controversial. The notion is that it causes harmful effects on the vasculature, but no study found the association in inflammatory states. Vitamin D deficiency is known to play an important role in autoimmune disease advancement. Deficiency further contributes to ED progression and atherosclerosis formation. Vitamin D supplements have beneficial effects on the overall RA state. Certain genotypes are prone to CV disease formation. ABI<0.9 index is specific to peripheral obstruction but not sensitive enough. The addition of Doppler spectral waveform analysis or exercise-induced ABI will detect the disease more accurately. ABI>1.3 is indicative of arterial incompressibility, which is a distinct process, with various risk factors. It is associated with cortical bone loss. Young to middle-aged patients or ones with early-stage (<5 years) disease were found to have a higher risk of ED than the healthy population. It should be noted that in contrast to normal peripheral arterial morphological parameters, there is increased arterial stiffness in central vascular beds in short-term disease. The processes in microvascular or macrovascular beds are independent or partially dependent, thus separate assessment would have benefits. Arteriosclerosis detection by PWV and Aix is informative and straightforward. PWV is assumed to be a gold standard, while Aix can be an add-on parameter. Reverse wave pressure calculating separately from Aix demonstrates more accurate and reliable results. Longitudinal, prospective analyses are needed to evaluate people with the highest risk of future arterial complications. Studies comparing diagnostic options are needed to choose the most appropriate and reliable ones.

## References

[1] Stevens RJ, Douglas KM, Saratzis AN, Kitas GD Inflammation and atherosclerosis in rheumatoid arthritis. Expert Rev Mol Med.

[2] Liang KP, Liang KV, Matteson EL, McClelland RL, Christianson TJ, Turesson C (2006). Incidence of noncardiac vascular disease in rheumatoid arthritis and relationship to extraarticular disease manifestations. Arthritis Rheum.

[3] Mori H, Okada Y, Kawaguchi M (2019). A study of the vascular endothelial function in patients with Type 2 diabetes mellitus and rheumatoid arthritis. Intern Med.

[4] Alkaabi JK, Ho M, Levison R, Pullar T, Belch JJ (2003). Rheumatoid arthritis and macrovascular disease. Rheumatology (Oxford).

[5] Roman MJ, Devereux RB, Schwartz JE (2005). Arterial stiffness in chronic inflammatory diseases. Hypertension.

[6] Fan F, Galvin A, Fang L (2014). Comparison of inflammation, arterial stiffness and traditional cardiovascular risk factors between rheumatoid arthritis and inflammatory bowel disease. J Inflamm.

[7] Geraldino-Pardilla L, Zartoshti A, Bag Ozbek A (2018). Arterial inflammation detected With 18 F-Fluorodeoxyglucose-Positron Emission Tomography in rheumatoid arthritis. Arthritis Rheumatol.

[8] Crowson CS, Rollefstad S, Ikdahl E (2018). Impact of risk factors associated with cardiovascular outcomes in patients with rheumatoid arthritis. Ann Rheum Dis.

[9] Pober JS, Sessa WC (2007). Evolving functions of endothelial cells in inflammation. Nat Rev Immunol.

[10] Alomari MA, Khabour OF, Alawneh K, Shammaa RA (2018). Possible modulation of vascular function measures in rheumatoid arthritis by homocysteine. Int J Rheumatol.

[11] Sandoo A, Hodson J, Douglas KM, Smith JP, Kitas GD (2013). The association between functional and morphological assessments of endothelial function in patients with rheumatoid arthritis: a cross-sectional study. Arthritis Res Ther.

[12] Hill CE, Phillips JK, Sandow SL (2001). Heterogeneous control of blood flow amongst different vascular beds. Med Res Rev.

[13] Tehan PE, Stewart S, Chuter VH, Carroll M, Rutherfurd KJ, Brenton-Rule A (2019). Relationship between lower limb vascular characteristics, peripheral arterial disease and gait in rheumatoid arthritis. Int J Rheum Dis.

[14] Rosano C, Newman AB, Katz R, Hirsch CH, Kuller LH (2008). Association between lower digit symbol substitution test score and slower gait and greater risk of mortality and of developing incident disability in well-functioning older adults. J Am Geriatr Soc.

[15] Shovman O, Tiosano S, Comaneshter D, Cohen AD, Amital H, Sherf M (2016). Aortic aneurysm associated with rheumatoid arthritis: a population-based cross-sectional study. Clin Rheumatol.

[16] Henke PK, Sukheepod P, Proctor MC, Upchurch GR Jr, Stanley JC (2003). Clinical relevance of peripheral vascular occlusive disease in patients with rheumatoid arthritis and systemic lupus erythematosus. J Vasc Surg.

[17] Bergholm R, Leirisalo-Repo M, Vehkavaara S, Mäkimattila S, Taskinen MR, Yki-Järvinen H (2002). Impaired responsiveness to NO in newly diagnosed patients with rheumatoid arthritis. Arterioscler Thromb Vasc Biol.

[18] Kim YS, Sung YK, Choi CB (2012). The major determinants of arterial stiffness in Korean patients with rheumatoid arthritis are age and systolic blood pressure, not disease-related factors. Rheumatol Int.

[19] Chuang YW, Yu MC, Lin CL (2016). Risk of peripheral arterial occlusive disease in patients with rheumatoid arthritis. A nationwide population-based cohort study. Thromb Haemost.

[20] Gunter S, Robinson C, Norton GR (2017). Cardiovascular risk factors and disease characteristics are consistently associated with arterial function in rheumatoid arthritis. J Rheumatol.

[21] Gkaliagkousi E, Anyfanti P, Chatzimichailidou S (2018). Association of nocturnal blood pressure patterns with inflammation and central and peripheral estimates of vascular health in rheumatoid arthritis. J Hum Hypertens.

[22] Gerli R, Schillaci G, Giordano A (2004). CD4+CD28- T lymphocytes contribute to early atherosclerotic damage in rheumatoid arthritis patients. Circulation.

[23] Li P, Han CX, Ma CL (2013). Determinants of brachial-ankle pulse wave velocity in Chinese patients with rheumatoid arthritis. Clin Dev Immunol.

[24] Vaudo G, Marchesi S, Gerli R (2004). Endothelial dysfunction in young patients with rheumatoid arthritis and low disease activity. Ann Rheum Dis.

[25] Roldán JF, Escalante A, del Rincón I (2008). Impaired arterial function associated with thinning of cortical bone in rheumatoid arthritis. Arthritis Rheum.

[26] Hong Q, Xu J, Xu S, Lian L, Zhang M, Ding C (2014). Associations between serum 25-hydroxyvitamin D and disease activity, inflammatory cytokines and bone loss in patients with rheumatoid arthritis. Rheumatology (Oxford).

[27] Caraba A, Crişan V, Romoşan I, Mozoş I, Murariu M (2017). Vitamin D status, disease activity, and endothelial dysfunction in early rheumatoid arthritis patients. Dis Markers.

[28] Erre GL, Piga M, Fedele AL (2018). Prevalence and determinants of peripheral microvascular endothelial dysfunction in rheumatoid arthritis patients: a multicenter cross-sectional study. Mediators Inflamm.

[29] Verma I, Syngle A, Krishan P (2017). Predictors of endothelial dysfunction and atherosclerosis in rheumatoid arthritis in Indian population. Indian Heart J.

[30] Türk SM, Cansu DÜ, Teke HÜ (2018). Can we predict thrombotic tendency in rheumatoid arthritis? A thromboelastographic analysis (with ROTEM). Clin Rheumatol.

[31] Sandoo A, Carroll D, Metsios GS, Kitas GD, Veldhuijzen van Zanten JJ (2011). The association between microvascular and macrovascular endothelial function in patients with rheumatoid arthritis: a cross-sectional study. Arthritis Res Ther.

[32] Ristić GG, Subota V, Lepić T (2015). Subclinical atherosclerosis in patients with rheumatoid arthritis and low cardiovascular risk: the role of von Willebrand factor activity. PLoS One.

[33] Seitz C, S S, Berens N, Bröcker E, -B -B, Trautmann A (2010). Leg ulceration in rheumatoid arthritis - an underreported multicausal complication with considerable morbidity: analysis of thirty-six patients and review of the literature. Dermatology.

[34] Mangoni AA, Baghdadi LR, Shanahan EM (2017). Methotrexate, blood pressure and markers of arterial function in patients with rheumatoid arthritis: a repeated cross-sectional study. Ther Adv Musculoskelet Dis.

[35] Das S, Mohanty M, Padhan P (2015). Outcome of rheumatoid arthritis following adjunct statin therapy. Indian J Pharmacol.

[36] Mäki-Petäjä KM, Day L, Cheriyan J (2016). Tetrahydrobiopterin supplementation improves endothelial function but does not alter aortic stiffness in patients with rheumatoid arthritis. J Am Heart Assoc.

[37] Verhoeven F, Prati C, Maguin-Gaté K, Wendling D, Demougeot C Glucocorticoids and endothelial function in inflammatory diseases: focus on rheumatoid arthritis. Arthritis Res Ther.

[38] del Rincón I, O'Leary DH, Haas RW, Escalante A (2005). Effect of glucocorticoids on the arteries in rheumatoid arthritis [published correction]. Arthritis Rheum.

[39] Dimitroulas T, Hodson J, Sandoo A, Smith J, Kitas GD (2017). Endothelial injury in rheumatoid arthritis: a crosstalk between dimethylarginines and systemic inflammation. Arthritis Res Ther.

[40] Dimitroulas T, Sandoo A, Hodson J, Smith JP, Kitas GD (2016). In vivo microvascular and macrovascular endothelial function is not associated with circulating dimethylarginines in patients with rheumatoid arthritis: a prospective analysis of the DRACCO cohort. Scand J Clin Lab Invest.

[41] Gonzalez-Juanatey C, Testa A, Garcia-Castelo A (2003). HLA-DRB1 status affects endothelial function in treated patients with rheumatoid arthritis. Am J Med.

[42] Rac-Albu M, Iliuta L, Guberna SM, Sinescu C (2014). The role of ankle-brachial index for predicting peripheral arterial disease. Maedica (Buchar).

[43] Yang Y, Wang Z, Fu Z (2019). Stiffening of aorta is more preferentially associated with rheumatoid arthritis than peripheral arteries. Rheumatol Int.

[44] Grech AC, Gatt A, Borg AA, Formosa C (2017). Determining the presence of peripheral arterial disease in patients with rheumatoid arthritis. Mediterr J Rheumatol.

[45] Vitalis A, Lip GY, Kay M, Vohra RK, Shantsila A (2017). Ethnic differences in the prevalence of peripheral arterial disease: a systematic review and meta-analysis. Expert Rev Cardiovasc Ther.

[46] del Rincón I, Haas RW, Pogosian S, Escalante A (2005). Lower limb arterial incompressibility and obstruction in rheumatoid arthritis. Ann Rheum Dis.

[47] Hughes AD, Park C, Davies J (2013). Limitations of augmentation index in the assessment of wave reflection in normotensive healthy individuals. PLoS One.

[48] Gunter S, Robinson C, Woodiwiss AJ (2018). Arterial wave reflection and subclinical atherosclerosis in rheumatoid arthritis. Clin Exp Rheumatol.

[49] Stamatelopoulos KS, Kitas GD, Papamichael CM (2010). Subclinical peripheral arterial disease in rheumatoid arthritis. Atherosclerosis.

[50] Dzieża-Grudnik A, Sulicka J, Strach M (2017). Arterial stiffness is not increased in patients with short duration rheumatoid arthritis and ankylosing spondylitis. Blood Press.

